# Effect of the healthy school recognized campus initiative on metabolic syndrome among adolescents in texas: a pilot randomized factorial trial study

**DOI:** 10.1186/s41043-026-01261-6

**Published:** 2026-02-14

**Authors:** Mia H. Putnam, Gabrielli T. de Mello, Julie Gardner, Alexandra L. MacMillan Uribe, Chad D. Rethorst, Allyson Schaefers, Rebecca A. Seguin-Fowler, Ryan W. Walters, Jacob Szeszulski

**Affiliations:** 1 School of Public Health, Texas A&M University, 212 Adriance Lab Rd, College Station, Texas, 77843 TX USA; 2 Institute for Advancing Health Through Agriculture (IHA), Texas A&M AgriLife Research, 17360 Coit Rd, Dallas, 75252 TX USA; 3 Texas A&M AgriLife Extension, 600 John Kimbrough Boulevard, College Station, Texas, 77843 TX USA; 4 Texas 4-H Youth Development, 1470 William D Fitch Parkway, College Station, Texas, 77845 TX USA; 5 Institute for Advancing Health Through Agriculture (IHA), Texas A&M AgriLife Research, 498 Olsen Drive, College Station, Texas, 77845 TX USA; 6 Department of Nutrition, College of Agriculture & Life Sciences, Texas A&M University, 498 Olsen Drive, TX 77845 College Station, United States; 7 Department of Clinical Research and Public Health, Creighton University, 7710 Mercy Road Educ Bldg, Ste 502, Omaha, 68124 NE USA

**Keywords:** Physical activity, Exercise, Healthy eating, Obesity, Cardiovascular disease

## Abstract

**Background:**

School-based interventions are effective in improving physical activity and healthy eating in adolescents. However, there has been limited research into how bundled school-based programs, and their components, may improve adolescents’ metabolic health. The purpose of this study is to evaluate the effectiveness of the Healthy School Recognized Campus (HSRC) – a bundled school-based health initiative – and two HSRC components on metabolic syndrome (MetS) and other obesity-related risk factors.

**Methods:**

A 2 × 2 cluster randomized factorial trial was conducted in eight HSRC middle schools (*n* = 107 students) to pilot test a novel program’s (Strong Teens for Healthy Schools [STHS]) and implementation strategy’s (mentoring program) preliminary effects on MetS z-scores, obesity-related risk factors (e.g., BMI z-scores), and behavioral risk factors (e.g., physical activity, healthy eating). General linear model frameworks were used to estimate the main and interaction effects on outcomes. Additionally, bivariate tests were run to determine if students had a significant change in outcomes over the year-long HSRC intervention.

**Results:**

Across all schools, students involved in the HSRC program decreased BMI z-scores (mean difference [MD]=-0.12 ± 0.33, *p*=.003); whereas students increased glucose levels (MD = 6.29 ± 18.34, *p*=.003), total MetS factors (MD = 0.23 ± 0.83, *p*=.015), and fruit and vegetable consumption (MD = 34.47 ± 92.33, p = < 0.001). The STHS intervention was associated with an increase in waist circumference (β = 5.55, 95%CI: 0.14, 10.96, *p*=.044). The mentoring program was associated with a reduction in systolic blood pressure (β=-6.32, 95%CI: -12.13, -0.51, *p*=.033) and increase in dermal carotenoids (β = 65.50, 95%CI: 18.11, 112.89, *p*=.007).

**Conclusions:**

HSRC improved some obesity-related risk factors, but not MetS severity. The STHS intervention independently increased waist circumference, and the mentoring program independently improved dermal carotenoid levels and systolic blood pressure. Results suggest that complex interaction effects exist in bundled interventions within school settings; however, bundled EBPs may be effective in reducing some obesity-related outcomes (e.g., BMI z-scores) over a one-year duration for adolescents in East Texas.

**Supplementary Information:**

The online version contains supplementary material available at 10.1186/s41043-026-01261-6.

## Introduction

Adolescence is a critical period during which health behaviors and lifestyle choices are established [1]. School-based interventions are a common setting for health promotion interventions for adolescents as over 65 million youth attend schools for an average of 6.5 h per day [2, ]. When schools deliver evidence-based programs (EBPs), they allow a large population of adolescents to be engaged in physical activity and healthy eating for extended periods of time, which increases the likelihood that EBPs can improve cardiovascular health outcomes [3, 4]. EBPs have been found to be effective in school settings at reducing the prevalence of overweight adolescents by up to 8% [5]. Additionally, when school based EBPs improve physical activity and healthy eating environments, they may be more effective than EBPs that only address student’s behaviors [–]. 

Texas A&M AgriLife’s Healthy School Recognized Campus (HSRC) initiative promotes the delivery of physical activity and healthy eating programs by bundling multiple school-based EBPs. The use of bundled EBPs may improve health outcomes better than a single evidence-based intervention (EBI) alone [8]. For a school to be recognized as a HSRC, they are required to host a school-wide walking challenge to increase students’ physical activity (i.e., Walk Across Texas), at least one other AgriLife program for students, and at least one AgriLife program for adults [9], which are delivered by extension agents (i.e., health educators) [9, 10]. However, the HSRC initiative has yet to be evaluated as it is pragmatically implemented in a real-world setting where bundled EBPs are being concurrently delivered.

Bundled EBPs also present additional challenges to the assessment of study outcomes. For example, the use of multiple interventions, with different ecological levels and behavioral targets (e.g., physical activity vs. nutrition vs. both), can have variable effects on students’ health and behavioral outcomes [11]. Bundling EBPs can also create implementation barriers, both for individuals implementing the programs and at the organizational level (e.g., additional complexity, more resources required) [12]. For example, previously cited barriers to implementing physical activity and nutrition programs include student’s perception of healthy food, availability of healthy foods and physical activity opportunities, and cost, among others [–13]. Implementation strategies (i.e., methods to improve program delivery), such as peer-to-peer mentoring programs, that address these barriers can help to improve the delivery of bundled interventions [14]. 

The primary aim of this study was to (Aim 1) pilot test an approach where we systematically varied and evaluated variations in one program (Strong Teens for Healthy Schools [STHS]) and one implementation strategy (peer-to-peer mentoring) [descriptions below] as part of HSRC. Specifically, we were interested if HSRC, and/or involvement of a school in the mentoring implementation strategy, the STHS program, or both, significantly decreased adolescent metabolic syndrome (MetS) severity and/or physical activity and healthy eating behaviors over one school year. As HSRC has not been evaluated as a bundled approach, we also (Aim 2) evaluated the pre- and post-test effectiveness of HSRC on different metabolic and obesity-related outcomes over a school year. Furthermore, given the differences in health behaviors (i.e. average steps per day, fruit and vegetable consumption, etc.) between boys and girls, we (Aim 3) compared the results by student sex.

## Methods

### Study design

We piloted a 2 × 2 cluster randomized factorial trial with a pre- (T1; August to October of 2022) and post-test (T2; April to May of 2023) evaluation in eight East Texas middle schools. Each school received a $3,500 stipend, materials, and support from extension agents (i.e., health educators) to deliver the HSRC initiative. Extension agents were selected based on the county where the school was located. Schools completed a form selecting their programs for the year, signed a memorandum of understanding agreeing to participate, and signed a site authorization allowing data collection to occur at their school. At the end of each year, Extension agents reported the programs that the school implemented to meet HSRC’s requirements. Parents’ consent and student’s assent was also obtained. This study was approved by the IRB at Texas A&M University (IRB2022-0800).

### Study conditions

Each of the eight middle schools received the HSRC initiative; however, using a simple random assignment procedure, schools were assigned to receive, or not receive, two different HSRC components at a 1-to-1 ratio (Table 1). As the factorial design was constructed to be balanced across conditions, four of the middle schools were randomized to each program/implementation strategy, and four did not receive them. This type of design also increases the likelihood of balance across groups on known (e.g., other randomized conditions) and unknown confounders (e.g., variation in the way programs are delivered). Conditions included:

**Table 1:** Experimental conditions for the 2^2^ factorial design

School 1 (*n*=16)	Healthy Schools Recognized Campus (HSRC) Initiative	Peer-to-Peer Mentoring (Mentoring) – Implementation Strategy	Strong Teens for Healthy Schools (STHS) - Program
	Yes	Yes	Yes
School 3 (*n*=13)	Yes	Yes	Yes
School 6 (*n*=16)	Yes	Yes	No
School 7 (*n*=5)	Yes	Yes	No
School 5 (*n*=21)	Yes	No	Yes
School 4 (*n*=5)	Yes	No	No
School 8 (*n*=12)	Yes	No	Yes
School 2 (*n*=18)	Yes	No	No

- **Condition 1 – Mentoring (Implementation Strategy)**: Extension agents who delivered HSRC met four times over the course of the year (January, February, March, and April) for 60 min per meeting, to talk about where they were in the implementation process, successes, challenges, as well as plans for long-term implementation of HSRC. Agents were also encouraged to share resources with one another (e.g., flyers, materials). The meetings were designed to increase knowledge about HSRC, brainstorm solutions for overcoming barriers, and increase self-efficacy for successfully completing HSRC. A research team member was trained to lead all meetings.
- **Condition 2 – Strong Teens for Healthy Schools (Program)**: STHS is a multilevel, theory-based, and 24-session (1 h each) program delivered by extension agents or school staff that focuses on physical activity, healthy eating, positive youth development, and cardiovascular disease prevention. Throughout STHS, students identify and advocate for a sustainable change in the school health environment (e.g., installation of a school garden). At the individual-level, STHS uses the theory of planned behavior to provide education about physical activity and healthy eating [15, 16]. At the social level, STHS activities focus on developing support for other students who are engaging in physical activity and healthy eating by creating positive changes to the school environment [17, 18]. Finally, at the school-level, the WSCC model identifies ten domains that are relevant for students’ health, and small groups of students consider each domain, and each group picks one for their STHS project [19–21]. Extension agents completed one hour of in-person training on STHS and completed fidelity checklists after each session.

### Data collection

*Participants* As not all Extension educators have the capacity to provide education in multiple languages, inclusion criteria were: (1) 6th −8th grade student and (2) the student being able to speak, write, and read in English. Exclusion criteria were: (1) taking a medication that affected weight, (2) enrolled in a weight loss program up to 3 months before the pre-test data collection phase, and (3) pregnant or up to 3-months post-partum. Parents signed consent forms in English or Spanish, and students provided written assent before data collection. All students were allowed to participate in the HSRC activities, regardless of whether they consented/assented for the study.

At T1 and T2, students completed a survey, point of care blood test, and physical examination performed by trained researchers. Researchers completed two one-hour training sessions prior to conducting the assessments. The 20-minute paper survey was used to collect demographic information (e.g., age, student-reported sex, student-reported race/ethnicity, and grade level) and other physical activity and healthy eating constructs. The point-of-care blood test, using the CardioChek Plus analyzer (PTS Diagnostics; Whitestown, Indiana), was used to collect total cholesterol (mg/dL), HDL-cholesterol (mg/dL), triglycerides (mg/dL), and glucose (mg/dL). Emails were sent to parents to remind students to avoid food and drink before testing; however, several students did not come to school fasted. Still, as data collection occurred on a single day, blood outcomes and fasting status were collected on all students. Finally, physical examination data was collected using standardized written protocols, including for measuring waist circumference using a Gulick measuring tape (i.e., midway between the bottom of the ribs and the top of the hip bone); systolic blood pressure (SBP), diastolic blood pressure (DBP), and pulse (beats per minute) using an automated Omron sphygmomanometer (i.e., right arm after five minutes seated); height using a stadiometer; and weight using a Tanita body composition analyzer (Tanita US; Arlington Heights, Illinois). Averages of height (in), weight (lb), waist circumference (cm), SBP (mmHg), DBP (mmHg), body fat percentage, and dermal carotenoid level were calculated by taking the two closest values out of the three measurements and averaging them. Additional behavioral measures were also taken after physical examination, including dermal carotenoids (i.e., estimate of fruit and vegetable consumption) using Veggie Meter^®^ (Longevity Link Corporation; Salt Lake City, UT) and average steps per day (over a 5-day period during school hours) using FitBits^®^ (Google FitBit; San Francisco, CA). The Veggie Meter is a tool to estimate fruit and vegetable intake and has been previously validated for adolescents [22]. Students received items (e.g., duffle bags, t-shirts) worth up to $40 at each data collection timepoint (T1 and T2), and students received the items even if all data collection measurements were not completed.

### MetS & risk factors

MetS is a cluster of interconnected risk factors that are associated with an increased risk of chronic diseases (e.g., cardiovascular disease, obesity, etc.) [23, 24]. For MetS risk and the MetS risk components, each of the variables were coded as dichotomous (if students had that risk component based on MetS criteria) and continuous (z-scores for MetS and BMI; raw values for other risk factors). MetS and its risk components were assessed using the International Diabetes Federation (IDF) adolescent (10–15 years) definition (i.e., waist circumference risk and at least two of the four other risk components) [25, 26]. The students 90th percentile waist circumference (cm) cutoff was based off the international waist circumference percentile cutoffs, with specific cutoffs for each age and sex [27]. The MetS risk components are:

- Waist circumference (cm) *≥* 90th percentile.
- Triglycerides *≥* 150 mg/dL.
- HDL-cholesterol < 40 mg/dL.
- SBP *≥* 130 mmHg or DBP *≥* 85 mmHg.
- Fasting blood glucose *≥* 100 mg/dL.

### MetS Z-scores

MetS z-scores and BMI z-scores were calculated as a continuous measure of severity of MetS and BMI, respectively [27]. To obtain a MetS z-score and BMI z-score, demographic information (date-of-birth, date of appointment [to ascertain age], sex, and race), height and weight, and the MetS components (SBP, HDL-cholesterol, triglycerides, and glucose levels) were entered into a z-score calculator [27]. Age was calculated by subtracting date-of-birth with standardized pre-test (September 1, 2022) and the post-test (May 31, 2023) dates.

### Data analysis

Data was cleaned and evaluated using STATA Version 18 (StataCorp LLC; College Station, TX) using per-protocol analysis. Across all schools, bivariate analyses (e.g., paired t-tests) were conducted to evaluate differences in outcomes between T1 and T2 over one-year of participation in HSRC. Sex-stratified bivariate analyses were also performed. For our primary outcome (i.e., MetS z-score), each MetS risk factor (continuous), and other obesity-related outcomes that had significant change in the bivariate analysis, multivariable models were used to estimate the effects of the strategies (mentoring, STHS, or the interaction) on those outcomes. Due to minimal clustering of variance between schools (ICC < 0.001), we used a single-level general linear model (ANCOVA), as opposed to a linear mixed-effects model. In Model 1, we added the mentoring strategy (dichotomous, dummy coded), STHS intervention (dichotomous, dummy coded), and the interaction between Mentoring and STHS as predictors, and the outcome at T1 as covariates. Model 2 included the same predictors and outcomes as Model 1 along with age (continuous, centered at 12 years) and sex (dichotomous) as covariates. As several students were not fasted at T1 (*n* = 20) and T2 (*n* = 26), a sensitivity analysis was performed for blood glucose (Table S1). As results did not vary if students were consistently fasted or unfasted, both the fasted and non-fasted students (*n* = 79) were used for the main and stratified analyses. Given that this was a pilot study, we calculated effect sizes and 95% confidence intervals for all outcomes.

## Results

Students (*n* = 114) from 8 schools in East Texas consented to participate in the study (Figure S1). On average, schools completed 2.88 ± 0.83 (range 2–4) youth programs and 1.50 ± 0.93 adult programs (range 0–3). All mentoring sessions occurred as planned. Only three out of four schools completed STHS. Seven students were removed from the study for not completing any T1 or T2 measures (Figure S1). Of the 107 valid students (8 schools with an average of 13.3 students/school), the mean (standard deviation) age was approximately 12.3 ± 0.9 years (between 10 and 14 years old), 51.9% were female, and students were in the 6th (31.4%), 7th (42.9%) and 8th grade (25.7%) (Table S2) at baseline. Students were predominately white (68.9%) and Hispanic (28.3%). At T1, the prevalence rate for MetS was 10.3% (*n* = 88); at T2, the prevalence rate was 9.1% (*n* = 88) (Table S3). There did not appear to be any differences between baseline demographic characteristics of students across schools (Table S4).

### Intervention effects of mentoring and STHS

Participation in mentoring (*n* = 51), STHS (*n* = 63), nor the combination of them (*n* = 30) had a statistically significant effect on MetS z-scores (Table 2). Students who were in the mentoring-only strategy compared to students not in mentoring or STHS (control group) had a non-significant decrease in SBP at T2 (β= −5.87, 95%CI: −12.04, 0.30, *p*=.062) in Model 1, but did have a significant decrease in Model 2 (i.e., when controlling for age and gender of students) (β= −6.32, 95%CI: −12.13, −0.51, *p*=.033). Additionally, students in the mentoring-only group had a significant increase in dermal carotenoids in Model 1 (β = 67.22, 95%CI: 19.73, 114.70, *p*=.006) and Model 2 (β = 65.50, 95%CI: 18.11, 112.89, *p*=.007) compared to students in neither mentoring or STHS. Furthermore, participation in the STHS-only group compared to no participation in mentoring or STHS was related to a significant increase in waist circumference (cm) in both Model 1 (β = 5.81, 95%CI: 0.42, 11.20, *p*=.035) and Model 2 (β = 5.55, 95%CI: 0.14, 10.96, *p*=.044). Finally, the interaction of mentoring and STHS had a significant effect on dermal carotenoids in both Model 1 (β= −73.48, 95%CI: −135.05, −11.91, *p*=.020) and Model 2 (β= −72.14, 95%CI: −133.34, −10.95, *p*=.021). For the outcomes of triglycerides, HDL-C, glucose, and DBP, and BMI z-scores none of the strategies nor the interaction had a significant effect.

**Table 2:** General linear multivariable model (ANCOVA) results for metabolic syndrome Z-Scores, metabolic syndrome Components, and other metabolic/Behavioral risk factor outcomes

		Model 1			Model 2		
**Outcome**	**Predictors**	**Coefficient**	**p**	**95% CI**	**Coefficient**	**p**	**95% CI**
MetS Z-scores at T2 (*n* = 77)	Mentoring	0.08	0.616	−0.23, 0.38	0.04	0.787	−0.26, 0.34
	Strong Teens	0.09	0.533	−0.20, 0.38	0.06	0.697	−0.23, 0.34
	Mentoring*Strong Teens	−0.11	0.583	−0.53, 0.30	−0.05	0.818	−0.45, 0.36
Waist Circumference at T2 (cm) (*n* = 99)	Mentoring	1.07	0.721	−4.87, 7.01	1.25	0.676	−4.66, 7.16
	Strong Teens	5.81	**0.035**	0.42, 11.20	5.55	**0.044**	0.14, 10.96
	Mentoring*Strong Teens	−4.07	0.307	−11.94, 3.80	−4.06	0.304	−11.86, 3.74
Triglycerides at T2 (mg/dL) (*n* = 79)	Mentoring	27.49	0.061	−1.29, 56.27	28.15	0.059	−1.15, 57.45
	Strong Teens	4.60	0.746	−23.63, 32.84	4.90	0.740	−24.39, 34.18
	Mentoring*Strong Teens	−8.53	0.675	−48.92, 31.86	−11.00	0.600	−52.61, 30.61
HDL-Cholesterol (mg/dL) (*n* = 79)	Mentoring	1.15	0.514	−2.34, 4.64	1.24	0.485	−2.28, 4.76
	Strong Teens	−1.57	0.358	−4.95, 1.81	−1.37	0.431	−4.82, 2.08
	Mentoring*Strong Teens	1.04	0.663	−3.69, 5.76	1.05	0.662	−3.72, 5.83
SBP at T2 (mmHg) (*n* = 98)	Mentoring	−5.87	0.062	−12.04, 0.30	−6.32	**0.033**	−12.13, −0.51
	Strong Teens	0.08	0.976	−5.38, 5.54	0.02	0.994	−5.19, 5.23
	Mentoring*Strong Teens	5.26	0.197	−2.78, 13.30	5.27	0.170	−2.29, 12.83
DBP at T2 (mmHg) (*n* = 98)	Mentoring	1.17	0.599	−3.23, 5.56	1.17	0.604	−3.30, 5.64
	Strong Teens	3.44	0.086	−0.50, 7.38	3.64	0.077	−0.41, 7.69
	Mentoring*Strong Teens	−1.23	0.671	−6.96, 4.50	−1.23	0.673	−7.03, 4.56
Glucose at T2 (mg/dL) (*n* = 79)	Mentoring	5.43	0.319	−5.35, 16.21	5.03	0.340	−5.41, 15.46
	Strong Teens	8.71	0.101	−1.74, 19.15	8.69	0.095	−1.55, 18.93
	Mentoring*Strong Teens	−11.20	0.131	−25.80, 3.41	−9.62	0.180	−23.78, 4.55
BMI Z-scores at T2 (*n* = 77)	Mentoring	0.01	0.958	−0.22, 0.24	0.01	0.907	−0.22, 0.25
	Strong Teens	−0.13	0.239	−0.34, 0.09	−0.13	0.238	−0.35, 0.09
	Mentoring*Strong Teens	−0.05	0.768	−0.35, 0.26	−0.06	0.692	−0.37, 0.25
Dermal Carotenoids at T2 (*n* = 97)	Mentoring	67.22	**0.006**	19.73, 114.70	65.50	**0.007**	18.11, 112.89
	Strong Teens	1.61	0.940	−41.00, 44.22	−4.60	0.832	−47.45, 38.26
	Mentoring*Strong Teens	−73.48	**0.020**	−135.05, −11.91	−72.14	**0.021**	−133.34, −10.95

Model 1: adjusted for outcome at baseline (T1), Model 2: adjusted for outcome at baseline (T1), age (centered at 12 years), and sex (female); Bolded values are *p*<.05; p=p-value; 95%CI= 95% Confidence Interval for coefficient; MetS=Metabolic Syndrome; SBP=Systolic Blood Pressure; DBP=Diastolic Blood Pressure; BMI=Body Mass Index

### Pre- and post-intervention effects of HSRC

There were no differences in MetS z-scores between T1 and T2 during participation in HSRC (Table 3). BMI z-scores significantly decreased (mean difference [MD]= −0.12, 95%CI: −0.19, −0.04, *p*=.003); Table 3). Average dermal carotenoid levels (MD = 34.47, 95%CI: 15.86, 53.08, p = < 0.001), glucose (MD = 6.29 mg/dL, 95%CI: 2.18, 10.40, *p*=.003), and total MetS risk factors (MD = 0.23, 95%CI: 0.05, 0.42, *p*=.015) all significantly increased. There was a non-significant increase in average steps per day (MD = 325.07, 95%CI: −22.67, 672.81, *p*=.066).

**Table 3:** Paired sample T-Test results for continuous outcomes from the overall sample (*n*=107)

	T1	T2	Paired Samples t-test (T2-T1)				
	M ± SD	M ± SD	MD ± SD	t	df	*p*	d (95% CI)
**Metabolic Syndrome**							
Metabolic Syndrome Z-Scores	0.08 ± 0.76	0.14 ± 0.75	0.06 ± 0.46	1.14	76	0.256	0.13 (−0.09, 0.35)
Total MetS Risk Factors	0.90 ± 1.07	1.13 ± 1.09	0.23 ± 0.83	2.48	76	**0.015**	0.28 (0.06, 0.51)
**Metabolic Syndrome Risk Factors**							
Average Waist Circumference	76.75 ± 13.17	75.76 ± 16.16	−0.99 ± 9.65	−1.02	98	0.311	−0.10 (−0.30, 0.10)
Triglycerides (mg/dL)	75.11 ± 32.40	84.91 ± 46.30	9.80 ± 46.09	1.89	78	0.063	0.21 (−0.01, 0.44)
HDL-cholesterol (mg/dL)	48.52 ± 9.58	48.81 ± 9.72	0.29 ± 5.39	0.48	78	0.632	0.05 (−0.17, 0.27)
Average Systolic Blood Pressure (mmHg)	107.09 ± 9.14	105.95 ± 10.92	−1.14 ± 10.80	−1.04	97	0.299	−0.11 (−0.30, 0.09)
Average Diastolic Blood Pressure (mmHg)	61.69 ± 8.23	60.61 ± 7.69	−1.09 ± 8.64	−1.25	97	0.216	−0.13 (−0.32, 0.07)
Glucose (mg/dL)	95.67 ± 11.50	101.96 ± 16.32	6.29 ± 18.34	3.05	78	**0.003**	0.34 (0.12, 0.57)
**Other Metabolic Risk Factors**							
Total Cholesterol (mg/dL)	119.87 ± 20.39	120.05 ± 23.78	0.18 ± 22.11	0.07	78	0.943	0.01 (−0.21, 0.23)
Calculated LDL (mg/dL)	56.33 ± 18.94	54.26 ± 23.30	−2.07 ± 23.33	−0.79	78	0.432	−0.09 (−0.31, 0.13)
Average Pulse (beats/minute)	78.56 ± 10.53	80.06 ± 13.02	1.70 ± 12.13	1.39	97	0.169	0.14 (−0.06, 0.34)
BMI Z-Scores	0.85 ± 1.00	0.73 ± 1.01	−0.12 ± 0.33	−3.05	76	**0.003**	−0.35 (−0.58, −0.12)
BMI	22.05 ± 4.67	22.22 ± 5.07	0.17 ± 1.36	1.23	98	0.223	0.12 (−0.07, 0.32)
Average Body Fat (%)	24.86 ± 9.57	25.40 ± 9.52	0.54 ± 4.61	1.14	94	0.256	0.12 (−0.08, 0.32)
**Behavioral Risk Factors**							
Average Dermal Carotenoid	244.91 ± 84.06	279.38 ± 85.63	34.47 ± 92.33	3.68	96	**<0.001**	0.37 (0.17, 0.58)
Average Standardized Steps per Day	4837.23 ± 1647.40	5162.30 ± 1579.15	325.07 ± 1403.37	1.87	64	0.066	0.23 (−0.01, 0.48)

Bolded values are *p*<.05 (two-sided). T1=Pre-Test; T2=Post-Test; MD=Mean difference (T2-T1); SD=Standard deviation; M=Mean; df=Degrees of freedom; p=p-value; d=Cohen’s d effect size; 95%CI= 95% confidence interval

### Sex stratified effects of HSRC

Male students (*n* = 51) had significant increases in MetS z-scores (MD = 0.18, 95%CI: 0.03, 0.33, *p*=.021) and glucose (MD = 11.76, 95%CI: 4.77, 18.74, *p*=.002) (Table 4). Male students also had significant decreases in BMI z-scores (MD= −0.15, 95%CI: −0.24, −0.05, *p*=.003), average waist circumference (MD= −3.06 cm, 95%CI: −5.86, −0.26, *p*=.033), and calculated LDL cholesterol (MD=−6.23 mg/dL, 95%CI: −12.29, −0.16, *p*=.044). Female students (*n* = 55) had significant decreases in average systolic blood pressure (MD=−3.4 mmHg, 95%CI: −6.01, −0.79, *p*=.012) and an increase in dermal carotenoids (MD = 40.70, 95%CI: 15.48, 65.91, *p*=.002).

**Table 4:** Sex stratified paired sample T-Tests results for metabolic syndrome & metabolic syndrome risk factors

	Male					Female				
	T1	T2				T1	T2			
	M ± SD	M ± SD	df	p	d (95% CI)	M ± SD	M ± SD	df	p	d (95% CI)
**Metabolic Syndrome**										
MetS Z-Scores	0.07 ± 0.63	0.25 ± 0.70	36	**0.021**	0.40 (0.06, 0.73)	0.08 ± 0.87	0.03 ± 0.79	39	0.492	−0.11 (−0.42, 0.20)
Total MetS Risk Factors	0.78 ± 0.82	1.05 ± 1.03	36	**0.039**	0.35 (−0.02, 0.68)	1.00 ± 1.26	1.20 ± 1.16	39	0.160	0.23 (−0.09, 0.54)
**Metabolic Syndrome Risk Factors**										
Average Waist Circumference (cm)	76.92 ± 12.21	73.86 ± 17.09	47	**0.033**	−0.32 (−0.61, −0.03)	76.60 ± 14.14	77.56 ± 15.18	50	0.465	0.10 (−0.17, 0.38)
Triglycerides (mg/dL)	67.57 ± 18.20	76.27 ± 29.93	36	0.076	0.30 (−0.03, 0.63)	81.76 ± 40.12	92.52 ± 56.26	41	0.232	0.19 (−0.12, 0.49)
HDL-cholesterol (mg/dL)	47.22 ± 8.30	48.16 ± 8.48	36	0.282	0.18 (−0.15, 0.50)	49.67 ± 10.55	49.38 ± 10.77	41	0.738	−0.05 (−0.35, 0.25)
Average Systolic Blood Pressure (mmHg)	108.82 ± 10.81	110.04 ± 11.30	47	0.482	0.10 (−0.18, 0.39)	105.43 ± 6.90	102.03 ± 9.02	49	**0.012**	−0.37 (−0.66, −0.08)
Average Diastolic Blood Pressure (mmHg)	60.11 ± 7.69	60.09 ± 7.62	47	0.987	−0.00 (−0.29, 0.28)	63.21 ± 8.53	61.10 ± 7.81	49	0.093	−0.24 (−0.52, 0.04)
Glucose (mg/dL)	95.49 ± 9.80	107.24 ± 20.37	36	**0.002**	0.56 (0.21, 0.91)	95.83 ± 12.92	97.31 ± 9.74	41	0.506	0.10 (−0.20, 0.41)
**Other Risk Factors**										
Total Cholesterol (mg/dL)	117.78 ± 19.40	114.24 ± 18.01	36	0.198	−0.22 (−0.54, 0.11)	121.71 ± 21.28	125.17 ± 27.09	41	0.392	0.13 (−0.17, 0.44)
Calculated LDL (mg/dL)	57.05 ± 17.14	50.83 ± 17.71	36	**0.044**	−0.34 (−0.67, −0.01)	55.70 ± 20.57	57.28 ± 27.16	41	0.703	0.06 (−0.24, 0.36)
Average Pulse	75.48 ± 9.32	78.17 ± 13.27	47	0.116	0.23 (−0.06, 0.52)	81.12 ± 10.96	81.87 ± 12.64	49	0.677	0.06 (−0.22, 0.34)
BMI Z-Scores	0.83 ± 0.97	0.68 ± 0.98	36	**0.003**	−0.52 (−0.86, −0.18)	0.87 ± 1.03	0.78 ± 1.05	39	0.149	−0.23 (−0.55, 0.08)
BMI	21.49 ± 4.49	21.54 ± 4.87	47	0.745	0.05 (−0.24, 0.33)	22.58 ± 4.82	22.85 ± 5.22	50	0.194	0.18 (−0.09, 0.46)
Average Body Fat (%)	20.84 ± 8.96	22.03 ± 8.72	44	0.178	0.20 (−0.09, 0.50)	28.48 ± 8.69	28.43 ± 9.25	49	0.911	−0.02 (−0.29, 0.26)
**Behavioral Risk Factors**										
Average Dermal Carotenoids	256.50 ± 76.85	284.07 ± 80.53	45	0.057	0.29 (−0.01, 0.58)	234.45 ± 89.53	275.15 ± 90.58	50	**0.002**	0.45 (0.17, 0.74)
Standardized Steps per Day	5338.43 ± 1852.74	5756.63 ± 1646.08	33	0.153	0.25 (−0.09, 0.59)	4287.52 ± 1190.31	4510.45 ± 1224.73	30	0.250	0.21 (−0.15, 0.57)

Bolded values are *p*<.05 (two-sided). T1=Pre-Test; T2=Post-Test; MD=Mean difference (T2-T1); SD=Standard deviation; M=Mean; df=Degrees of freedom; p=p-value; d=Cohen’s d effect size; 95%CI= 95% confidence interval

## Discussion

Overall, this study found that a school’s involvement in HSRC improved some metabolic and obesity-related outcomes for students, but not MetS severity. Specifically, students significantly reduced their BMI z-scores and increased dermal carotenoids (i.e., a marker of fruit and vegetable consumption) over the year they were involved in HSRC. The piloted implementation strategy (mentoring) and program (STHS) that we systematically varied also improved some outcomes but did not affect change in MetS severity. For example, students at the schools that received mentoring-only significantly decreased their average SBP and improved their dermal carotenoids compared to students who were at schools that did not receive mentoring or STHS. For schools that received STHS-only, students did not significantly improve any outcomes. Furthermore, the interaction between mentoring and STHS erased the positive effect of mentoring on dermal carotenoids, demonstrating that the interaction between these two components resulted in a worse outcome for dermal carotenoids than either component alone. Finally, the study also found that HSRC resulted in different effects for male and female students. Male students involved in HSRC significantly improved their BMI z-scores, waist circumference, and LDL-cholesterol, whereas female adolescents improved their SBP and dermal carotenoids.

The significant improvement in BMI z-scores and dermal carotenoids, and marginal improvements in steps per day during a year-long involvement in HSRC could indicate that HSRC is having the intended positive effects on physical activity and nutritional outcomes, which may be leading to improvements in weight profiles. However, the changes in weight were not enough to improve MetS risk over an approximate nine-month school year. One reason could be that a higher frequency and/or duration of the HSRC intervention may be needed to reduce MetS risk. Other reasons may include the small sample size and/or challenges obtaining a fasted sample for students. Additionally, the prevalence of MetS in this study was 10.2% at baseline, which reduced to 9.1% at follow-up. This could indicate that HSRC was acting as a protective factor for keeping MetS from increasing in the study population; however, future studies are needed to evaluate HSRC against a control condition. Still students were improving physical activity (steps per day), healthy eating (improved dermal carotenoid levels) and BMI z-scores, which is important, as these outcomes and MetS have been found to be positively associated with each other in multiple studies [28, 29]. These results are also consistent with other studies, which have found that school physical activity and healthy-eating interventions can improve behavioral outcomes (e.g., intakes of energy and macronutrients, engagement in physical activity) and reduce BMI z-scores [30, 31]. 

For the two HSRC program components that we systematically varied, we found that mentoring seemed to have a more significant impact on improving student’s outcomes. This finding is interesting, as the mentoring program is not likely to have a direct effect on the health outcomes of students. The mentoring program was designed to improve implementation outcomes for the HSRC programs (e.g., more programs delivered with high fidelity). However, if improved implementation led to better delivery of HSRC, better delivery could improve student outcomes, potentially via greater fidelity or a higher dose of HSRC [32–34]. However, we did not measure HSRC fidelity or dose as part of this study; thus, future studies should capture these data to evaluate the mediating effect of HSRC dose/fidelity on the relationship between the mentoring program and behavioral and health outcomes.

Schools that received the STHS program did not significantly improve any of our outcomes more than schools that received HSRC without STHS. In fact, at the end of the year, students in schools that received STHS increased their waist circumference more than students who did not receive STHS. STHS addresses many of the same behaviors (i.e., physical activity and nutrition) and health outcomes as other HSRC programs; thus, it does not appear to improve these outcomes more than any of the other programs within HSRC (e.g., Walk Across Texas), and related to waist circumference, it may not be as effective as HSRC for preventing abdominal obesity. Still, STHS could have a positive effect for schools that are not receiving HSRC and need physical activity and nutrition programs; however, this hypothesis should be evaluated via a randomized controlled trial against a suitable control condition. Additionally, when STHS was conducted in the same schools that received the mentoring program, it resulted in worse outcomes than either STHS or mentoring alone (i.e., antagonistic interaction effect). This finding suggests that complex interaction effects exist in bundled interventions, such as HSRC, and researchers and practitioners need to be cautious about adding or removing components from these types of interventions. Although, given the small sample size, both counterintuitive results should be interpreted with caution.

Finally, related to the sex-specific differences in outcomes, we found that neither male nor female students had a significant change in MetS severity. However, some outcomes improved for both males (BMI z-scores, waist circumference, and LDL-cholesterol) and females (SBP and dermal carotenoids). A larger cluster randomized trial of a physical activity intervention in elementary schools in Spain found that male students significantly decreased their waist circumference more than female students, whereas only female students had a significant decrease in LDL cholesterol and male students did not [35]. Although we are unclear why our study resulted in differential outcomes for males and females, we hypothesize that it could be related to differential engagement with activities that were a part of the programs – although more research would be needed to test this hypothesis. Furthermore, a meta-analysis of nonspecific and sex-specific interventions found that sex-specific interventions were moderately better at improving physical activity and healthy eating outcomes, but not adiposity outcomes [36]. Our study builds on these studies and several others by demonstrating that sex-specific differences can emerge even in non–sex-targeted interventions . Accordingly, it is important to identify which components of the interventions are leading to the sex-specific improvements in health. Once identified, we can tailor and deliver bundled interventions, including HSRC, to better meet the needs of male and female students.

One of the strengths of this study was the use of a factorial design. The factorial design allowed for random assignments of schools to answer multiple research questions (i.e., effectiveness of HSRC, impact of mentoring on outcomes, impact of STHS on outcomes, and interactions between mentoring and STHS). Another strength of this study was the collection of pre- and post-test data consistently among the eight middle schools. The protocols for assessing MetS, BMI, physical activity, and fruit and vegetable consumption worked well, and they can be replicated in future studies. However, more may need to be done to ensure students are fasting when point-of-care blood tests (i.e. glucose, cholesterol, and triglycerides) are collected in future studies. Finally, another strength of this study was that schools participating in this study were in a high-cardiovascular disease risk area, which allowed us to find a greater proportion of students with MetS (~ 9–10%) as compared to the general population (~ 3–4%). The higher proportion of students with MetS allowed for a better assessment of intervention effects on these outcomes.

One of the limitations of this study was that it was a short-term pilot study, and thus the sample size was relatively small (*n* = 107) and long-term sustainability was not assessed. To address this issue, effects sizes (Cohen’s d) were calculated. Related, the general linear models with age and sex included as predictors may be overfit. As overfitting can cause limited generalizability to the applicability of these models on future datasets with similar predictors, these results should be interpreted with caution. Outside of the Veggie Meter^®^, which estimates fruit and vegetable consumption, this analysis does not include other dietary outcomes. As diet quality plays an important role in weight and MetS risk, future studies should use more comprehensive measures of diet quality. Finally, non-fasting students were included in bivariate and multivariate analyses. Although parents of students were sent reminders to have their children fast, this did not always occur; thus, there could be some social desirability bias related to students reporting being fasted in our glucose outcomes. To address this issue, a sensitivity analysis was conducted, and we found that there was no difference between the fasting-only students and all students.

## Conclusion

This study evaluated the HSRC initiative in a pragmatic real-world setting. Neither MetS z-scores nor MetS prevalence had statistically significant improvements. However, BMI z-scores and average dermal carotenoid levels both significantly improved for students during school participation in HSRC. Thus, HSRC may be an effective initiative in promoting healthy eating and physical activity behaviors by bundling school-based EBPs for adolescents in East Texas. For the two randomized strategies/interventions, the mentoring strategy was successful in improving some outcomes, but an antagonistic interaction effect existed between mentoring and STHS. Additionally, sex-specific differences were found for male and female students that need further exploration. The results from this study can help to inform the development and testing of bundled, school-based interventions that improve weight and health behaviors.

## Supplementary Information

Supplementary Material 1.

Supplementary Material 2.

## Data Availability

The datasets used and/or analyzed during the current study are available from the corresponding author on reasonable request.

## Abbreviations

MetS: Metabolic Syndrome
HSRC: Healthy School Recognized Campus
STHS: Strong Teens for Healthy Schools
SBP: Systolic Blood Pressure
DBP: Diastolic Blood Pressure
IDF: International Diabetes Federation
BMI: Body Mass Index
HDL-C: High-Density Lipoprotein Cholesterol
