# Supplementary figures and images for: Effect of the healthy school recognized campus initiative on metabolic syndrome among adolescents in texas: a pilot randomized factorial trial study
Source: J Health Popul Nutr. 2026 Feb 14;45:97. doi: 10.1186/s41043-026-01261-6 (PMC13011626; doi:10.1186/s41043-026-01261-6)

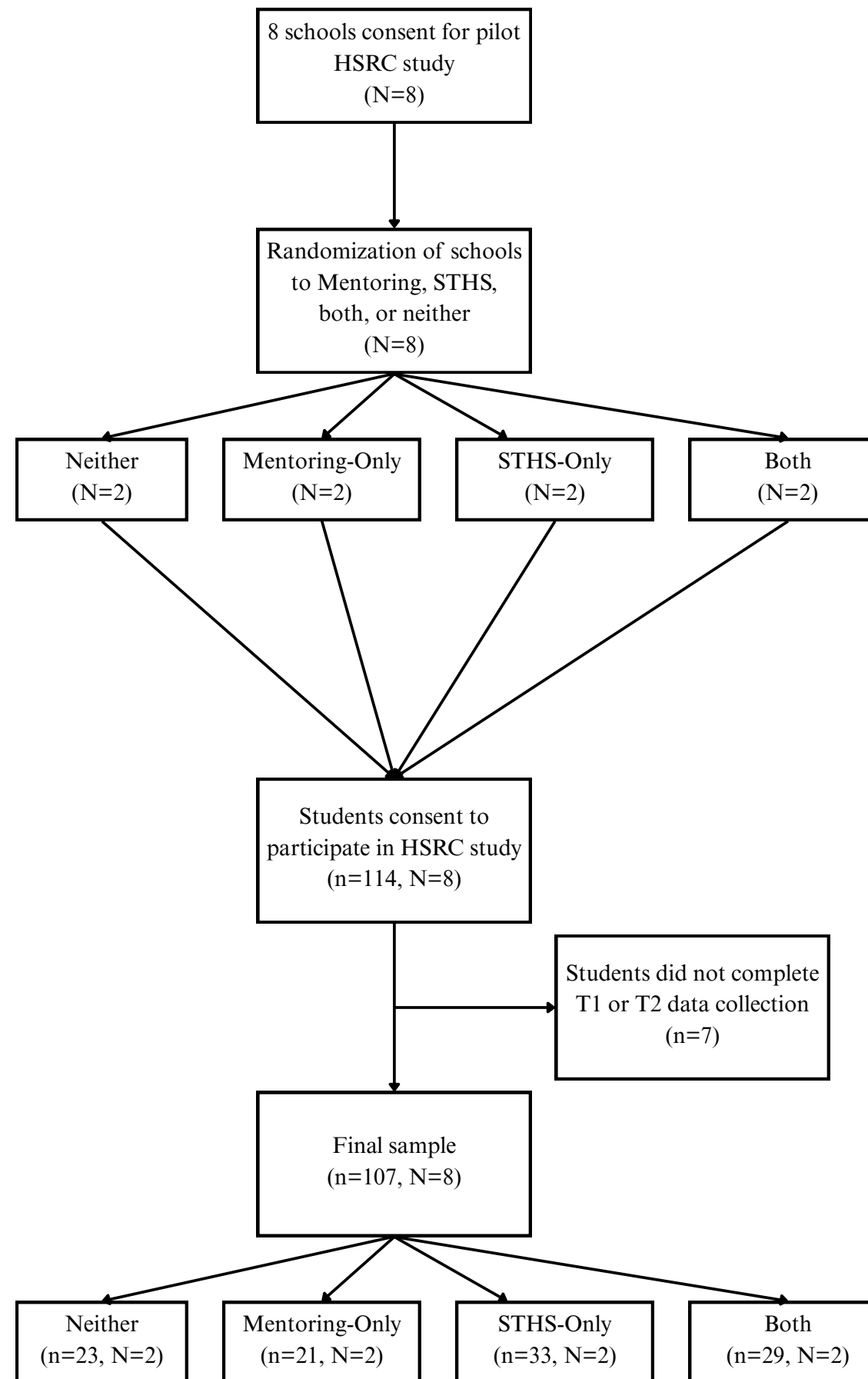

Supplement: Supplementary file 1 — Supplementary Material 1. [file 41043_2026_1261_MOESM1_ESM.pdf]
