# Supplementary material for: Effect of the healthy school recognized campus initiative on metabolic syndrome among adolescents in texas: a pilot randomized factorial trial study
Source: J Health Popul Nutr. 2026 Feb 14;45:97. doi: 10.1186/s41043-026-01261-6 (PMC13011626; doi:10.1186/s41043-026-01261-6)
Supplement: Supplementary file 2 — Supplementary Material 2. [file 41043_2026_1261_MOESM2_ESM.docx]

**Supplemental Material**

**Table S1.** Blood glucose sensitivity analysis for fasting-only vs. fasting/non-fasting

| Samples | Subsamples | Paired-sample t-tests for blood glucose (mg/dL) between T2 and T1 | | | |
| --- | --- | --- | --- | --- | --- |
|  |  | MD ± SD | df | p | d (95% CI) |
| Overall (n=107) | Fasting-only | 4.42 ± 12.43 | 42 | **.025** | 0.36 (0.05, 0.66) |
|  | Fasting & non-fasting | 6.29 ± 18.34 | 78 | **.003** | 0.34 (0.12, 0.57) |
| Male (n=51) | Fasting-only | 9.75 ± 11.42 | 15 | **.004** | 0.85 (0.28, 1.43) |
|  | Fasting & non-fasting | 11.76 ± 20.95 | 36 | **.002** | 0.56 (0.21, 0.91) |
| Female (n=55) | Fasting-only | 1.26 ± 12.10 | 26 | .593 | 0.10 (-0.27, 0.48) |
|  | Fasting & non-fasting | 1.48 ± 14.27 | 41 | .507 | 0.10 (-0.20, 0.41) |

*Note.* Bolded values are p<0.05 (two-sided); T1=Pre-Test; T2=Post-Test; MD=Mean Difference (T2-T1); df=degrees of freedom; p=p-value (two-sided); d=Cohen’s d effect size; 95% CI= 95% confidence interval.

**Table S2.** Demographics of Study Sample (n=107) at Baseline (T1)

| Variable | n (%) |
| --- | --- |
| **Age (in years), M±SD** | 12.28±0.86 |
| **Gender** |  |
| Male | 51 (48.11) |
| Female | 55 (51.89) |
| **Race/Ethnicity** |  |
| American Indian/Alaskan Native | 9 (8.49) |
| Asian | 4 (3.77) |
| Black or African American | 4 (3.77) |
| Native Hawaiian/Pacific Islander | 3 (2.83) |
| White | 73 (68.87) |
| Other | 29 (27.36) |
| Hispanic | 30 (28.30) |
| **Grade** |  |
| 6^th^ Grade | 33 (31.43) |
| 7^th^ Grade | 45 (42.86) |
| 8^th^ Grade | 27 (25.71) |

*Note.* M=Mean; SD=Standard deviation; n=subsample size; Race/Ethnicity variables were individual and not a singular categorical variable.

**Table S3.** Prevalence Rates of MetS by Demographic Variables Between Pre-Test and Post-Test

| Variable | | | Pre-Test (T1) | |  | Post-Test (T2) | |  |
| --- | --- | --- | --- | --- | --- | --- | --- | --- |
|  |  |  | n (%) with MetS | n (%) without MetS | Fischer’s Exact p-value | n (%) with MetS | n (%) without MetS | Fischer’s Exact p-value |
| **Total** | | | 9  (10.2%) | 79  (89.8%) | N/A | 8  (9.1%) | 80  (90.9%) | N/A |
| **Age at T1 (years)** | |  |  |  |  |  |  |  |
|  | <=10 | | 0  (0%) | 0  (0%) | 0.293 | 0  (0%) | 0  (0%) | 1.000 |
|  | 11-12 | | 7  (13.7%) | 44  (86.3%) |  | 5  (10.0%) | 45  (90.0%) |  |
|  | 13-14 | | 2  (5.4%) | 35  (94.6%) |  | 3  (7.9%) | 35  (92.1%) |  |
|  | 15-16 | | 0  (0%) | 0  (0%) |  | 0  (0%) | 0  (0%) |  |
| **Gender** | |  |  |  |  |  |  |  |
|  | Male | | 4  (9.3%) | 39  (90.7%) | 1.000 | 4  (9.9%) | 37  (90.2%) | 1.000 |
|  | Female | | 5  (11.1%) | 40  (88.9%) |  | 4  (8.5%) | 43  (91.5%) |  |
| **Race/Ethnicity** | |  |  |  |  |  |  |  |
|  | American Indian/Alaskan Native | | 0  (0%) | 6  (100%) | N/A | 0  (0%) | 6  (100%) | N/A |
|  | Asian | | 0  (0%) | 3  (100%) |  | 0  (0%) | 3  (100%) |  |
|  | Black or African American | | 0  (0%) | 4  (100%) |  | 0  (0%) | 4  (100%) |  |
|  | Native Hawaiian/  Pacific Islander | | 0  (0%) | 0  (0%) |  | 0  (0%) | 3  (0%) |  |
|  | White | | 4  (6.7%) | 56  (93.3%) |  | 4  (6.6%) | 57  (93.4%) |  |
|  | Other | | 6  (24.0%) | 19  (76.0%) |  | 5  (21.7%) | 18  (78.3%) |  |
|  | Hispanic | | 5  (20.8%) | 19  (79.2%) | .058 | 4  (16.0%) | 21  (84.0%) | .216 |
| **Grade Level** | |  |  |  |  |  |  |  |
|  | 6^th^ Grade | | 4  (14.8%) | 23  (85.2%) | .501 | 3  (12.0%) | 22  (88.0%) | .895 |
|  | 7^th^ Grade | | 4  (11.1%) | 32  (88.9%) |  | 3  (7.9%) | 35  (92.1%) |  |
|  | 8^th^ Grade | | 1  (4.2%) | 23  (95.8%) |  | 2  (8.3%) | 22  (91.7%) |  |

*Note.* n=subsample size. Percentages were calculated using row totals; Race/Ethnicity variables were individual and not a singular categorical variable.

**Figure S1.** Flow Diagram for School and Participant Inclusion


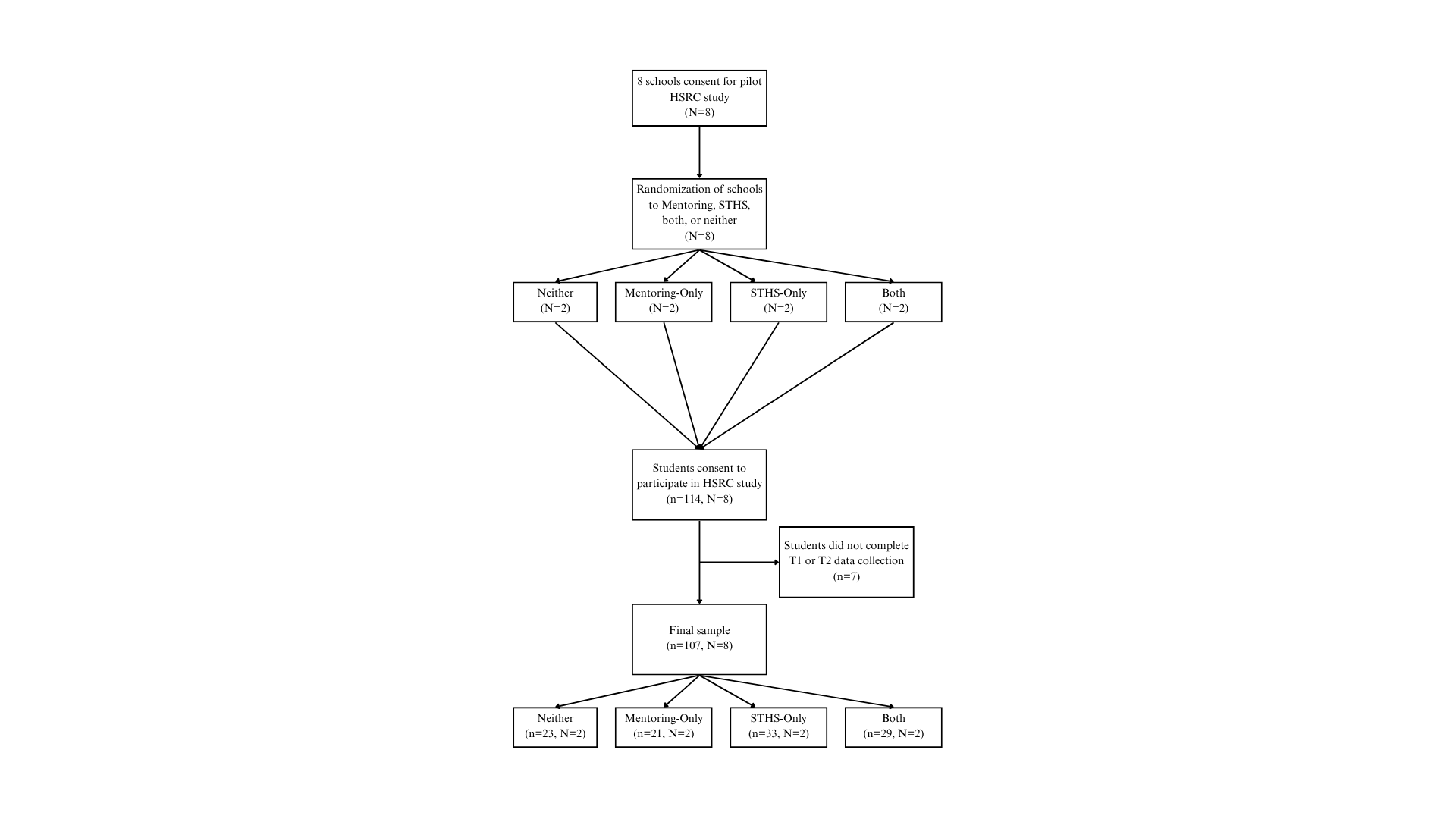


*Note*: This figure depicts the process for selecting schools and participants within those schools for inclusion for the HSRC Pilot study.

Table S4. Baseline Characteristics Between Schools

|  | | School 1  (n=16) | School 2 (n=18) | School 3 (n=13) | School 4 (n=5) | School 5 (n=21) | School 6 (n=16) | School 7 (n=5) | School 8 (n=12) |
| --- | --- | --- | --- | --- | --- | --- | --- | --- | --- |
| **Age (in years), M±SD** | | 12.81±0.54 | 11.56±0.78 | 11.92±0.76 | 13.4±0.55 | 12.29±0.78 | 12.25±0.86 | 12.4±0.89 | 12.58±0.67 |
| **Gender** | |  |  |  |  |  |  |  |  |
|  | Male | 8  (50.00%) | 10 (55.56%) | 6  (46.15%) | 1  (20.00%) | 8  (38.10%) | 9  (56.25%) | 2 (40.00%) | 7  (58.33%) |
|  | Female | 8  (50.00%) | 8  (44.44%) | 7  (53.85%) | 4  (80.00%) | 13  (61.90%) | 8  (43.75%) | 3 (60.00%) | 5  (41.67%) |
| **Race/Ethnicity** | |  |  |  |  |  |  |  |  |
|  | American Indian/Alaskan Native | 2  (12.50%) | 5  (27.78%) | 2  (15.38%) | 0  (0.00%) | 0  (0.00%) | 0  (0.00%) | 0  (0.00%) | 0  (0.00%) |
|  | Asian | 4  (25.00%) | 0  (0.00%) | 0  (0.00%) | 0  (0.00%) | 0  (0.00%) | 0  (0.00%) | 0  (0.00%) | 0  (0.00%) |
|  | Black or African American | 1  (6.25%) | 1  (5.56%) | 0  (0.00%) | 0  (0.00%) | 0  (0.00%) | 1  (6.25%) | 0  (0.00%) | 1  (8.33%) |
|  | Native Hawaiian/Pacific Islander | 1  (6.25%) | 2  (11.11%) | 0  (0.00%) | 0  (0.00%) | 0  (0.00%) | 0  (0.00%) | 0  (0.00%) | 0  (0.00%) |
|  | White | 11  (68.75%) | 15 (83.33%) | 13  (100%) | 5 (100.00%) | 2  (9.52%) | 14 (87.50%) | 4 (80.00%) | 9  (75.00%) |
|  | Other | 1  (6.25%) | 3  (16.67%) | 0  (0.00%) | 0  (0.00%) | 20  (95.24%) | 2  (12.50%) | 1 (20.00%) | 2  (16.67%) |
|  | Hispanic | 5  (31.25%) | 0  (0.00%) | 0  (0.00%) | 0  (0.00%) | 20  (95.24%) | 2  (12.50%) | 1 (20.00%) | 2  (16.67%) |
| **Grade** | |  |  |  |  |  |  |  |  |
|  | 6^th^ Grade | 0  (0.00%) | 14 (77.78%) | 6  (46.15%) | 0  (0.00%) | 6  (28.57%) | 5  (31.25%) | 2 (40.00%) | 0  (0.00%) |
|  | 7^th^ Grade | 9  (56.25%) | 3  (16.67%) | 5  (38.46%) | 0  (0.00%) | 10  (47.62%) | 7  (43.75%) | 0  (0.00%) | 11 (100.00%) |
|  | 8^th^ Grade | 7  (43.75%) | 1  (5.56%) | 2  (15.38%) | 5 (100.00%) | 5  (23.81%) | 4  (25.00%) | 3 (60.00%) | 0  (0.00%) |

*Note.* M=Mean; SD=Standard deviation; n=subsample size; Race/Ethnicity variables were individual and not a singular categorical variable.
